# Supplementary material for: Mapping the gene network landscape of Alzheimer’s disease through integrating genomics and transcriptomics
Source: PLoS Comput Biol. 2022 Feb 25;18(2):e1009903. doi: 10.1371/journal.pcbi.1009903 (PMC8906581; doi:10.1371/journal.pcbi.1009903)
Supplement: S1 Text — Supplementary Figures: Fig A: Network localization of AD GWAS genes. (A) AD gene network identified by GWAS. Gene colors represent the differential expression beta statistic in the temporal cortex between AD and healthy controls. Edges represent high confidence interactions in the STRING database. (B) Distribution of number of edges interconnecting AD GWAS genes (blue) or randomly selected gene sets (yellow). 80% of the AD GWAS genes were sampled 5000 times to create the distribution. Fig B: Heatmap of relative gene expression between AD patients and healthy control, temporal cortex. This figure shows the top 100 most differentially expressed genes. Note that the patients (columns) were not clustered here- they are sorted by healthy and AD status. Only the genes (rows) are clustered. Fig C: Transcriptomic study of AD genes in the temporal cortex. (A) Overlap of up-regulated genes in the Mayo Clinic RNAseq data and the expanded AD disease module. (B) Significant difference of Z-scores between up-regulated and the rest of genes. Fig D: Transcriptomic study of AD genes in the cerebellum. (A) No significant overlap of up-regulated genes in the Mayo Clinic RNAseq data and the expanded AD disease module. (B) No significant difference of Z-scores between up-regulated and the rest of genes. Fig E: Brain cell-specific mean expression (FPKM) of genes in identified clusters. Functional annotations for the clusters are 1: Immunoregulatory interactions between a lymphoid and non-lymphoid cell, 2: RNA metabolic process, 3: Complement activation, 4: Protein modification by small protein conjugation, 5: Clathrin-mediated endocytosis, 6: SNARE binding, 7: Regulation of plasma lipoprotein particle levels, 8: EPH-Ephrin signaling, 9: DNA-binding transcription factor activity, 10: Acetylcholine-gated cation-selected channel activity, 11: Interleukin-1 signaling, 12: GABAergic synapse. Fig F: Gene-enrichment analysis using predicted 646 proximity genes. The plot shows ratio of proximity [file pcbi.1009903.s001.docx]

**Supplemental Figures**


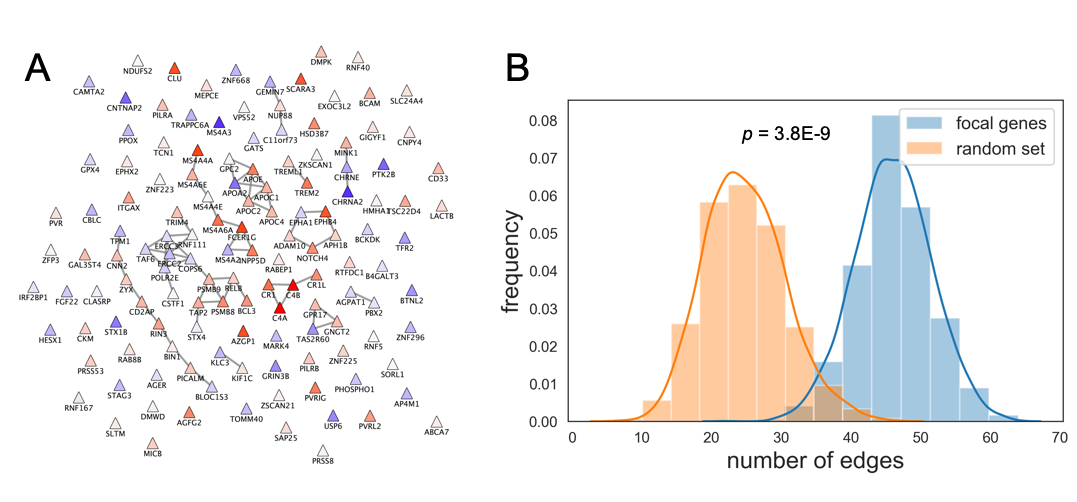


**Fig A: Network localization of AD GWAS genes**. (**A**) AD gene network identified by GWAS. Gene colors represent the differential expression beta statistic in the temporal cortex between AD and healthy controls. Edges represent high confidence interactions in the STRING database. (**B**) Distribution of number of edges interconnecting AD GWAS genes (blue) or randomly selected gene sets (yellow). 80% of the AD GWAS genes were sampled 5000 times to create the distribution.


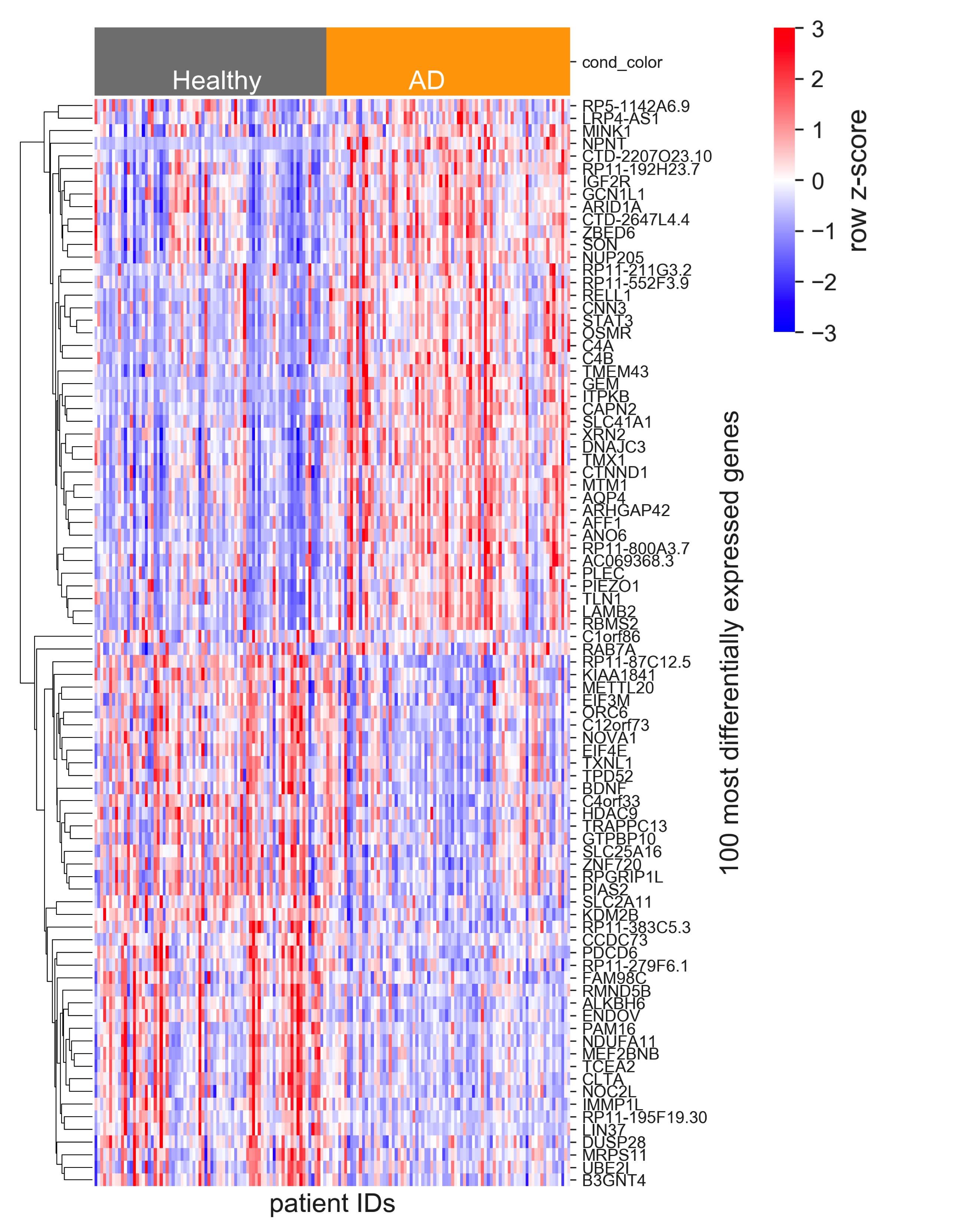


**Fig B: Heatmap of relative gene expression between AD patients and healthy control, temporal cortex.** This figure shows the top 100 most differentially expressed genes. Note that the patients (columns) were not clustered here- they are sorted by healthy and AD status. Only the genes (rows) are clustered.

**
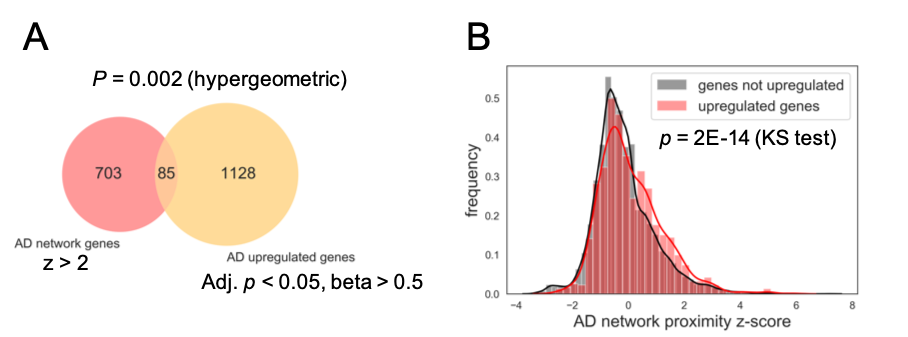
**

**Fig C: Transcriptomic study of AD genes in the temporal cortex.** (**A**) Overlap of up-regulated genes in the Mayo Clinic RNAseq data and the expanded AD disease module. (**B**) Significant difference of Z-scores between up-regulated and the rest of genes.


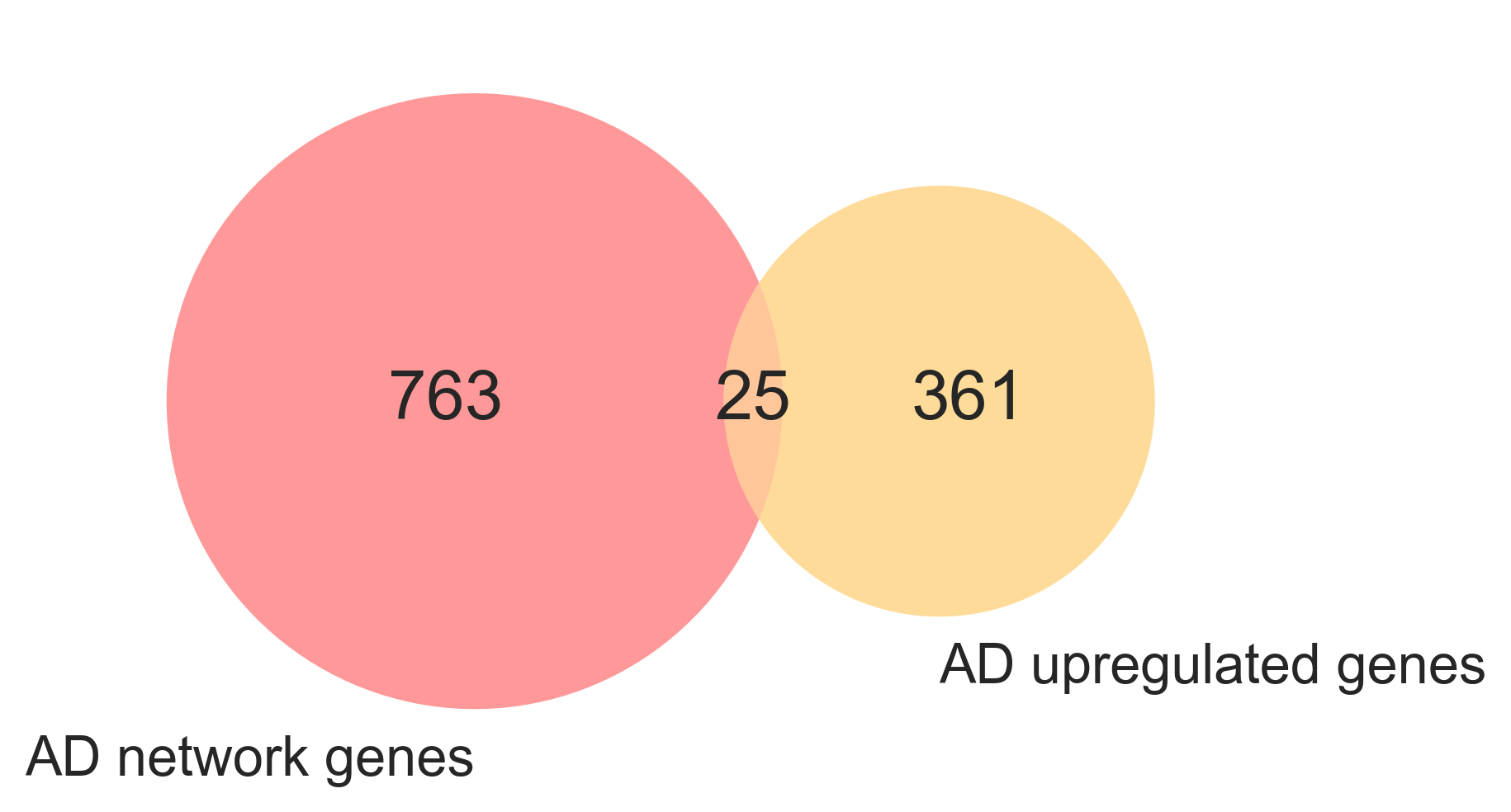

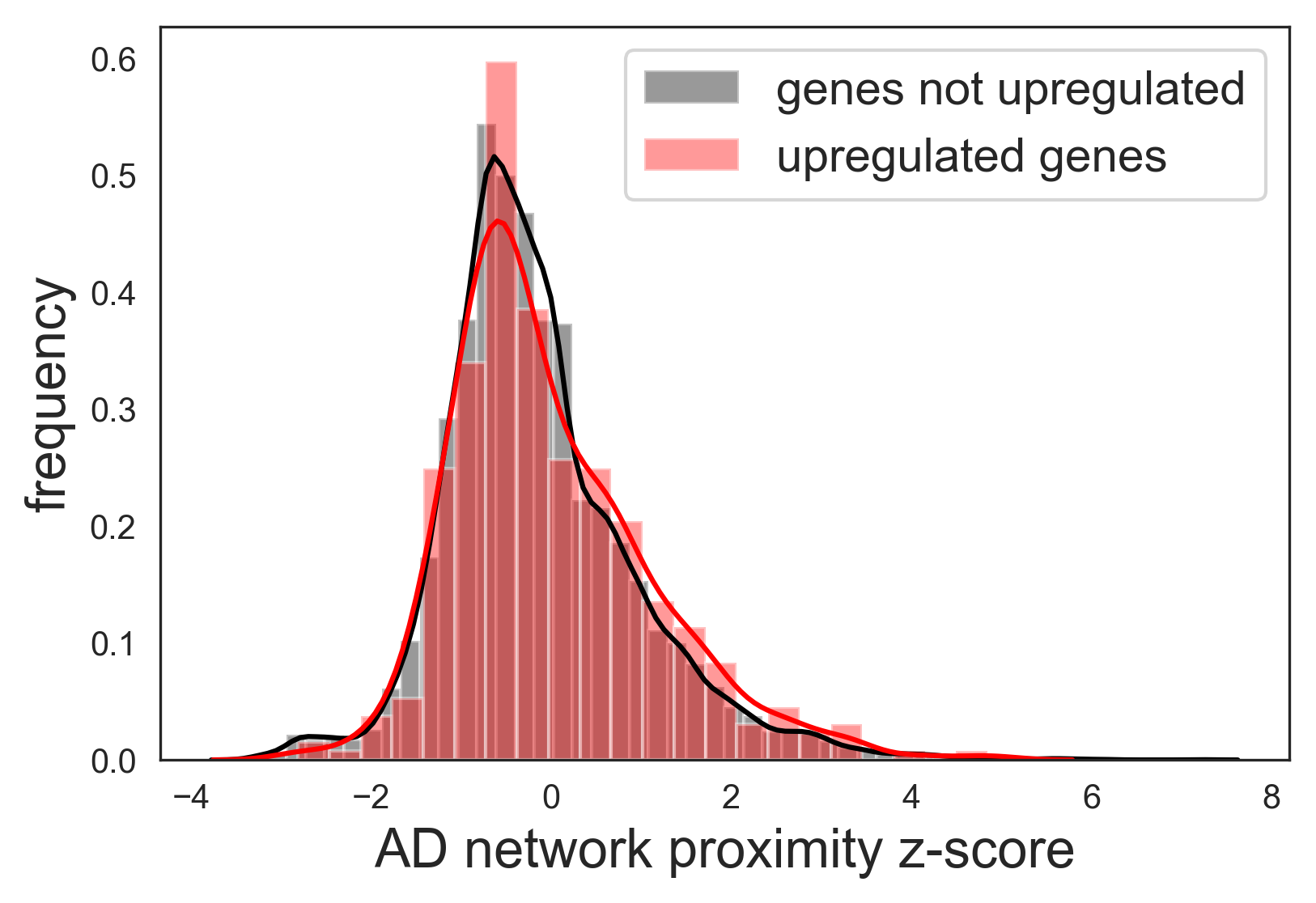


*p* = 0.069 (KS test)

*P* = 0.116 (hypergeometric)

A

B

**Fig D: Transcriptomic study of AD genes in the cerebellum.** (**A**) No significant overlap of up-regulated genes in the Mayo Clinic RNAseq data and the expanded AD disease module. (**B**) No significant difference of Z-scores between up-regulated and the rest of genes.

**
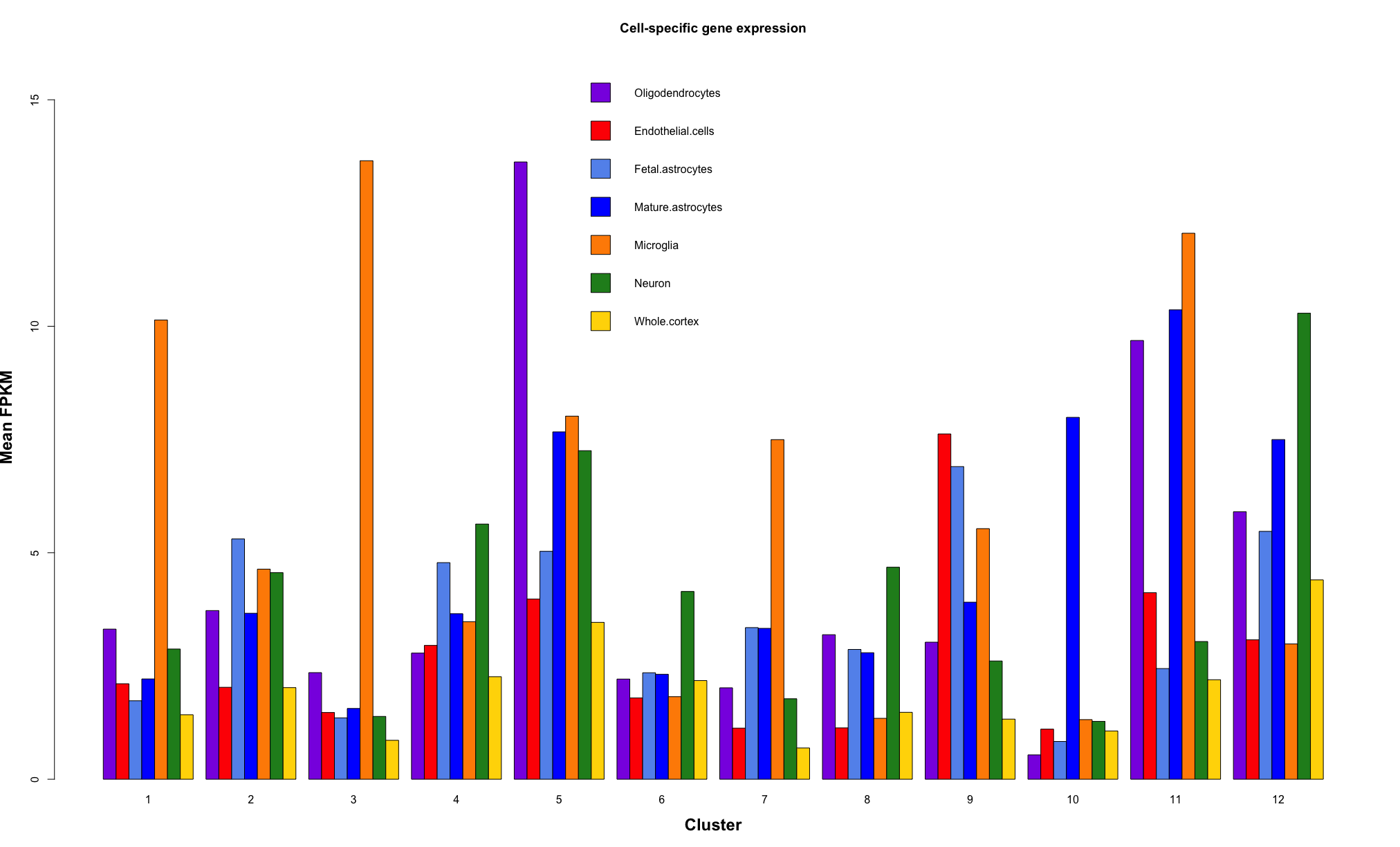
**

**Fig E: Brain cell-specific mean expression (FPKM) of genes in identified clusters.** Functional annotations for the clusters are 1: Immunoregulatory interactions between a lymphoid and non-lymphoid cell, 2: RNA metabolic process, 3: Complement activation, 4: Protein modification by small protein conjugation, 5: Clathrin-mediated endocytosis, 6: SNARE binding, 7: Regulation of plasma lipoprotein particle levels, 8: EPH-Ephrin signaling, 9: DNA-binding transcription factor activity, 10: Acetylcholine-gated cation-selected channel activity, 11: Interleukin-1 signaling, 12: GABAergic synapse.

**
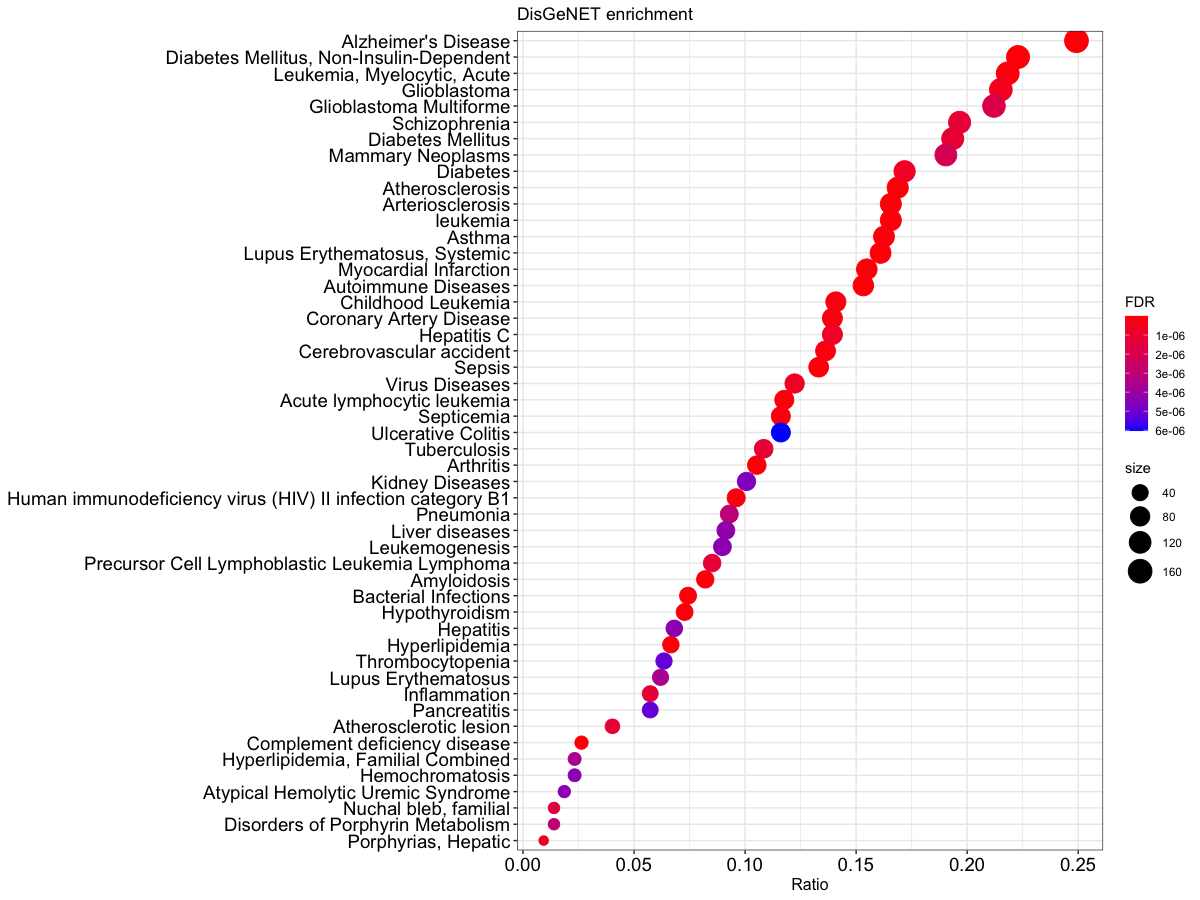
**

**Fig F: Gene-enrichment analysis using predicted 646 proximity genes.** The plot shows ratio of proximity genes overlapped with each disease-related gene set in the available databases.
