# Supplementary material for: Mapping the gene network landscape of Alzheimer’s disease through integrating genomics and transcriptomics
Source: PLoS Comput Biol. 2022 Feb 25;18(2):e1009903. doi: 10.1371/journal.pcbi.1009903 (PMC8906581; doi:10.1371/journal.pcbi.1009903)
Supplement: Attachment — Submitted filename: PLOS_Comput_Biol_response.docx [file pcbi.1009903.s003.docx]

**Reviewer #1**: The manuscript titled ‘Mapping the gene network landscape of Alzheimer’s disease through integrating genomics and transcriptomic’ (Manuscript No: PCOMPBIOL-D-21-01541) integrated multi-omics data to explore the key landscape of AD, which offer new insight for understanding the pathophysiology of Alzheimer’s disease. Especially, the starting point is based on the AD risk genes from GWAS analysis on a larger number of AD-by-proxy samples and autopsy-documented AD samples.
I have some points to discuss with authors:

Major comments:
1. The pathways analysis is based on the expanded network after network propagation algorithm by AD risk genes from GWAS analysis. As mentioned in introduction, genetic variations captured by GWAS are inherited, whereas transcriptomic provides information on factors that are both inherited and non-inherited.

1a. In table 1, it is interesting that the key pathways from expanded network module are not significant enriched when performing AD cortex RNAseq enrichment (BH FDR). The difference between genes from GWAS data and from transcriptomic data should be discussed. Or, further explanation could be given for that.

We appreciate the reviewer’s suggestion. We observed significant enrichment of dysregulated RNAseq genes in 8 clusters. We believe the following could be two main reasons: 1) we set a threshold for dysregulated genes as abs(beta)>0.5, which could have excluded GWAS-identified genes with smaller effects. 2) there could be more complicated epigenomic mechanisms that leads to significant differential expression of genes that were not shown in GWAS.

We’ve added the above in discussion:

***pp. 12, para. 2***: “…Significant enrichment of dysregulated genes in AD was observed in 8 out of the 17 annotated clusters. Possible reasons could lead to the discrepancies between the transcriptomic and GWAS data in the other 9 clusters, such as that genes with small effects were not consistently identified in the two datasets and/or there are more complicated epigenomic mechanisms involving differential expression of genes that were not detected by GWAS.”

The relevant sentences have been revised:

***pp. 24, para. 2***: “The significance of the overlap between dysregulated RNAseq genes and genes in each cluster was also assessed (hypergeometric test).”

***Table 1***: Revised the column name from “AD cortex RNAseq enrichment (BH FDR)” to “Enrichment of dysregulated genes in AD cortex (BH FDR)”.

1b. This work conducted network propagation algorithm based on AD risk genes from GWAS analysis and validate the obtained module clusters by significantly changed genes from transcriptomic data. What if network propagation algorithm was firstly performed on genes from transcriptomic data and then integrated with GWAS data? Will the final pathways and conclusions be different?

We thank the reviewer for this thoughtful comment. We established the network from GWAS-identified genes, because they represent inherited factors and are more stable as the backbone of the network. Transcriptomic genes were then overlaid on top of it, to cover the dynamic aspect of the disease, as they are both inherited and non-inherited. In our opinion, this order better resembles the natural pathology in the disease, which starts with inherited variations, and followed by non-inherited changes introduced during gene expression.

We will certainly consider the suggested approach in our future work. However, we believe it is beyond the scope of the present study, and we are concerned that presenting an alternative network would complicate the paper.

2. In section AD disease module of result, the manuscript declared that ‘Out of the 192 genes, 142 were found in the interactome (STRING database) and included in our subsequent analyses’. It means that 50 risk genes were excluded due to their missing in PPI network. However, it there possibility that some of 50 genes are the key genes to the pathophysiology of Alzheimer’s disease. If so, it might be one limitation of the work.
The authors had better provide which version of STRING database they utilized (The latest one or ?). In addition, the influence of some genes that cannot be mapped to interactome network should be discussed.

We appreciate the reviewer’s comment, and have added the list of the 50 excluded genes as **Supplementary Table S4**. As those genes are not included STRING interactome, they are likely less studied and difficult for functional analysis since less is known about their biological function. We agree with the reviewer that the possibility that these 50 genes are important to AD etiology can’t be ruled out, and therefore added it as a limitation of the present study.

***pp. 19, para. 2***: “In addition, the present study did not include 50 GWAS-identified genes that are not in the STRING interactome (Table S4). Their roles in AD etiology may also be important, and warrant further investigations.”

As stated in the methods section, we used High confidence human STRING interactome v10.5. “In our analysis we used interactions classified as ‘high confidence’ (combined score > 0.7), for the human interaction version 10.5” (***pp. 20, para. 2***).

Minor comments:
3. In the transcriptomic study in the section methods, to compare the overlapped situation between significant regulated genes from transcript data and the genes in expanded AD disease module, hypergeometric test was used. The total number samples considered in the test should be given. Is it the number of all the genes in GWAS analysis or all the genes in interactome network?

The total number of samples in the hypergeometric test was 14,631, which were the gene set found both in the interactome network and in the RNAseq data. We have added this information to the manuscript:

***pp. 24, para. 2***: “…using a total of 14,631 genes that were found both in the interactome network and in the RNAseq data.”

4. In figure 5, mean expression (FPKM) was used to show the cell-specific gene expression in each cluster. With gene expression data, GSEA (Gene set enrichment analysis) could also be considered to calculated the enrichment score of each cell type in specific clusters.

We appreciate the reviewer’s comment. We have performed a gene set enrichment analysis using the G:Profiler tool instead of GSEA on the gene set of individual clusters. These annotated pathways are listed in supplementary Table S2. We think a gene set enrichment analysis with GSEA will yield similar results thus may be repetitive. Also, the focus of our Fig. 5 aimed to present the overall gene expression across different cell types. However, we’ll be sure to consider GSEA in our future projects.

**Reviewer #2**: This paper integrated AD GWAS data with protein-protein interaction network and identified 142 AD risk genes plus 646 network-proximal genes. They also used independent data such as mouse knockout to validate the functions of those genes. Further, they related their PPI-based AD gene network to the population gene expression data (temporal cortex, AD vs. health), and found 17 gene clusters enriched with various functions/pathways. Using those enriched pathways, they also found a hierarchical structure in their AD network, implying higher order sequential interactions among AD gene functions. Finally, they associated the clusters with recent single cell data and found microglial functions in the clusters such as immunoregulation. In general, the paper reads logically and is also well organized. However, I have the following concerns and suggestions:

1. Under summary of molecular pathways of AD, the authors mention that the 5 classes of functionally related molecular pathways that are associated with AD could be changed slightly due to some pathways having multiple functions and being involved in various diseases. It may be helpful to indicate additional functions and how those 5 classes could change. Perhaps, providing this in supplementary information or elaborating a bit more could provide more informative context for the reader.

We’ve elaborated it with an example of the EPH-Ephrin signaling pathway.

***pp. 18, para. 1***: “An example is the EPH-Ephrin signaling pathway, which is associated both with synaptic dysfunction and CNS immune dysregulation.”

2. In Introduction, the authors do mention 4 papers (25 to 28) which potentially explain evidence of pathological progression and different regional vulnerabilities in AD, but it may be helpful to elaborate a bit more on whether those studies utilized the cerebellum as a hypothesized control and temporal cortex as major disease-relevant tissue. Perhaps adding some more literature and context on the brain region-specific progression of AD would help motivate this approach. Maybe, for example, the cerebellum is never impacted in AD (and a line on its functioning in the brain and statistics on why it is never involved), can be powerful to include. For instance, while it is great that the authors have done literature review for brain regions in AD, it may help to also synthesize and weave in that literature (as it may be too tedious for the reader to go through the literature to validate the support).

We have provided greater detail on our rationale for focusing on the temporal lobe and using the cerebellum as a control region, based on the known spread of tau pathology, and have added an additional citation on the spread of tau pathology in AD:

***pp. 5, para. 2***: “…based on the evidence that tau pathology, the pathological feature of AD that closely associated with clinical symptoms, emerges in temporal cortex early in the course of the disease before spreading to the other cortical areas in later stages, with minimal involvement to the cerebellum (25–29).”

3. In Introduction, it may help to add a parentheses definition of multiscale community detection to help the reader better understand.

We’ve revised the sentence to explain the context of multiscale community detection better:

***pp. 5, para. 2***: “Furthermore, application of multiscale community detection enables identification, annotation and mapping of the hierarchical substructure of biological pathways associated with the AD gene network (29).”

4. While the authors mention a lot of citations to support methodology (that is great and does boost confidence in the approaches), it may be helpful to elaborate on what “poorly characterized or new genes are”, especially since the authors removed such genes (please see AD disease module section, end of 1st paragraph, under results).

We removed 50 AD GWAS-identified significant genes that were not in STRING interactome. Those genes are either relatively new, or have not been well studied with regards to their biological functions. There is thus insufficient evidence to include these genes in our subsequent functional analysis and thus they were excluded.

We’ve added a list of these 50 removed genes in **Supplementary Table S4**.

5. From the manuscript, it is implied that 50 AD risk genes are excluded (that were previously identified in the large AD-by-proxy GWAS). Is there any other analysis that was done using these genes? Could there be other interactome data including these genes that may have been considered. What are the risks involved or cons of this approach? The authors only mention a very brief limitation in the discussion (i.e., “There are multiple choices of interactome, each with their own pros and cons, and many tissue-specific networks that can be obtained”). They could potentially add in details on losing the 50 AD risk genes using STRING interactome as a potential limitation. Could the genes have been uncovered in other interactomes? Elaborating more about the potential limitations can help as well and explaining more about the “pros and cons” of tissue-specific networks can help. In addition, authors may consider adding a few more details on why the STRING interactome was selected (i.e. what were the results of a systemic evaluation of the performance of diverse networks).

As stated in Potential Limitations (***pp. 19, para. 2***), we chose STRING interactome based a published systemic evaluation of different interactomes (***citation 70***), that concluded STRING as the most robust interactome. Therefore, we did not repeat this systemic evaluation in this current paper. Furthermore, we prefer a generic, non-tissue-specific interactome such as STRING for our first study using this approach for AD, as AD is affected by multiple systemic factors and comorbidities. In future work we will incorporate other tissue-specific interactome (such as GIANT as we also attempted here). Although some of the removed genes may be covered by other interactomes, those interactomes are either tissue-specific or underperformed in the systemic evaluation.

Please also see Reviewer #1, Point 2.

We’ve added the limitation about removing 50 genes:

***pp. 19, para. 2***: “In addition, the present study did not include 50 GWAS-identified genes that are not in the STRING interactome (Table S4). Their roles in AD etiology may also be important, and warrant further investigations.”

6. It is a bit confusing why the authors first use STRING interactome for the analysis (if only 142 AD GWAS risk genes are identified instead of the 191 that are identified in GIANT). Why replicate the analysis using GIANT to test consistency of the localization analysis and not the other way around, for example? Are the results of STRING in other applications much better than those of GIANT?

As mentioned above in response to point 5, the STRING interactome has been reported to be the most robust interactome. We also had a theoretical rationale for our approach, as we described iin response to Reviewer #1 Point 1b.

7. Under “Network propagation”, the authors mention that this process “simulates how heat would travel, through a network starting from a set of seed nodes.” What does heat represent (in terms of exploring the network proximity to a set of genes identified as significantly associated with AD), and why is this important?

Network propagation amplifies biological signals in networks, enabling exploration of genes which are significantly nearby in network space (improving on simpler measures such as first nearest neighbors). This method has been proven useful in numerous biological settings (Cowen et al. 2017. PMID: 28607512, DOI: 10.1038/nrg.2017.38). By simulating how heat would diffuse from a set of seed genes (seed genes in this case being those identified by GWAS), we are able to identify genes which are nearby to multiple seed genes, rather than just a single seed gene, since more heat will accumulate in genes which are close to multiple seed genes. Thus, we can identify genes which belong to the same biological pathway, since genes which are nearby in network space likely have similar biological functions. We have added more description to the text:

***pp. 20, para. 3***: “Network propagation amplifies biological signals in networks, enabling exploration of genes significantly nearby in network space, and improving on simpler measures such as first nearest neighbors. It simulates how heat would diffuse through a network starting from a set of GWAS-discovered seed nodes, which enables identification of genes proximal to multiple seed genes rather than a single one, because more heat will accumulate in genes that are close to multiple seed genes. It further assists in discerning genes in the same biological pathway, as the adjacent genes in a network likely have similar biological functions.”

8. In Discussion, the authors mention that the “dataset for AD risk genes from GWAS is incomplete”, but what does this mean and how can it be more complete?

We believe the list of AD risk genes are not complete, because the AD GWAS did not reach its full statistical power based on its sample size, so that the GWAS authors used AD-by-proxy cases to increase power. More significant genes with smaller effects may be detected by future larger GWAS.

9. In Fig. 5B, the authors mention that the inner circles are classes of identified pathways that are functionally related. What does the size of the inner circles exactly mean? It may be helpful to add a legend or a little more explanation to the figure caption. Also, if “Immune reactions” is right next to Microglia (which is expected as Microglia are immune cells in the brain) does this mean that it is only in Microglia (and same for Substance metabolism)? If that is the case, then about Endothelial cells as they don’t have 1 unique circle in front of them. What determines the size of each cell type in the outer circle? In other words, this “circos-like” plot can be a bit confusing and may need to be re-thought as it may currently be difficult for the reader to appreciate the novel findings. The main text only mentions 1 line for the figure “A schematic model of gene network in AD pathophysiology based on results of the present study is illustrated in Fig. 5B”, which does not explain much either.

We apologize for the confusion. **Fig. 5B** is a schematic but not quantitative plot, and the sizes of inner circles don’t represent the exact size of each class, but merely to show which classes are relatively bigger. It may thus not entirely accurate to say in the legend that circle sizes mean cluster sizes. We have revised the legend of Figure 5B as:

“Classes of identified pathways that are functionally related are presented as inner circles, with circle size roughly indicating relative class sizes…”

As genes in multiple clusters of “Immune reactions” and “Substance metabolism” were highly expressed in microglia, most of these two circles are overlaid by “Microglia”. Clusters in “Gene Transcription and RNA metabolism” are most expressed in both “Fetal astrocytes” and “Endothelial cells”, so the respective circle is overlaid by both cell types.

The length of a cell type label was set only for the ease of overlying relevant circles, and does not mean anything quantitatively.

10. In terms of visuals, Fig. 2 was superb in terms of detail, annotation, and legends, and innovation. Nonetheless, in the caption, the authors could add some example of proposed drug targets that have the bold black outlines. They could consider saying, (e.g. CHRNA2 and PTK2B are bolded examples). Also, what does the font size mean: why does CHRNA2 have a larger font size than TPM1 or ACTA2, for example? This explanation of font size can be added to Figure 2.

We thank the reviewer for the positive comments about Figure 2. As suggested, we have added examples in the figure legend of the AGORA proposed targets (“e.g. CHRNA2, PTK2B”). We have also added the explanation of the font sizes to the figure legend:

***Fig. 2 legend***: “… Seed genes or genes with AGORA targets are labeled with a larger font, while other genes identified by network propagation are labeled with a smaller font.”

11. Some of the other figures can be similar to Fig. 2 in terms of the approach. For instance, in Fig. 3, it may help to provide a legend (similar to the “cluster size” annotation in Fig. 2) to explain how the circle height relates to the # of genes that result in the phenotype when knocked out in mice. That is, for example, is the small circle just 1 gene and the large circle 100 genes? Fig. 4 caption explains pie chart colors but still having a legend (similar to Fig. 2 “DE Beta Statistic”) explaining the colors can help.

We’ve added more informative legends for Fig. 3 and 4, as suggested.

12. Some of the methods may need more explanation, especially in terms of the math. What is alpha in “Network propagation”. Where do we get the heat vector at time T (for F^t) and the value of Y? It may help if there is supporting code that is publicly-available for the readers to go through (i.e. an R markdown file or ipython notebook tutorial) to help them better understand how the network methods were exactly implemented.

We have added a Zenodo repository containing the code and jupyter notebooks needed to reproduce this analysis (https://zenodo.org/record/5786722#.Ybtti73MKC8 (DOI: 10.5281/zenodo.5786722)). We apologize for a formatting issue causing some of our variable definitions to disappear. This has now been fixed. Ft is a vector representing the heat contained in every node in the network at time t. Alpha, having a value between 0 and 1, is the fraction of total heat retained at every time step. We now refer the reader to the original publication where this method was developed, as well as to the jupyter notebooks for more information.

13. The authors also mention trends that are not statistically significant under the section “Cell-specific preferential expression”. It may help to report p-values to better understand if the results were close to statistical significance. Also, providing more biological interpretation of the cell-specific results would be helpful as the authors provide references to Figures and Supplements but could also list them out in the results. For instance, microglia have been recently implicated in AD neuroinflammatory processes.

We appreciate the reviewer’s suggestion, and have added respective *p*-values to the mentioned cell type-cluster pairs (***pp. 11, para. 2***).

As an exploratory network study that covers multiple aspects, we opted to provide biological interpretation of the significant cell-specific results in the respective subheadings of the Discussion (***pp. 14, para. 2-3***), as it is easier to include more details there compared to Results.

14. Under the statement: “We observed a cluster of genes associated with the ZAG-PIP complex”, it may be helpful to list out these genes that are involved in the ZAG-PIP complex. It appears as though ZAG-PIP complex is #15 module in Fig. 2 but there are no distinct clusters of genes there that point to it. The same for RNA metabolic process, which has no module of genes pointing to it but has some mention of genes like ZC3HAV1, DNAJC2, SYMPK, etc. in the Discussion section.

We apologize for the confusion. Please find the complete list of genes in each cluster (second column) in **supplementary Table S1** (for example, genes in partition/cluster 15). Due to limited space, we only illustrated the 5 largest clusters in **Fig. 2**, but not all the identified clusters.

15. It was great that the authors mention the different pathways in the Discussion section and the biology of the findings. It may be helpful to cite the Figures and supplementary figures associated with the different pathways. For instance, it is unclear where we could find the “cluster of 33 genes” identified by the data-driven approach.

We appreciate the reviewer’s suggestion and have added citations of the figures and tables in the relevant places in the discussion to improve clarity. Particularly, genes of each cluster can be found in **supplementary Table S1**, and pathways of each class are in **Table 1**.

***pp. 13, para. 1***: “Our data-driven approach identified a cluster of 33 genes (**Table S1**) …”

***pp. 14, para. 2***: “From the cell-specific data, we found genes in this cluster highly expressed in microglial cells (**Fig. 5A**).”

***pp. 14, para. 3***: “The crucial role that microglia-related immunoregulation plays in AD is supported by our network analyses and the preferential expression of these genes in microglia (**Fig. 5A**) …”

***pp. 15, para. 1***: “We observed a cluster of genes associated with the ZAG-PIP complex (**Table S1**) …”

***pp. 15, para. 3***: “In our disease module, *APOE* was located within a cluster of 49 genes (**Table S1**) …”

***pp. 16, para. 2***: “… majority of which were significantly down-regulated in AD (**Table S1**).”

***pp. 16, para. 3***: “… including post-translational modifications, intracellular trafficking, vesicular transport and degradation of protein (**Table 1**).”

16. The authors mention Fig 4A under Proteostasis, but there is no figure 4A.

We apologize for the typo. It has been corrected to “**Fig. 5A**”.

17. The organization of the discussion is unclear, nonetheless. The authors mention potentially clinically-relevant clusters to discuss, and it appears as though those could belong to Fig. 2, but the authors do not really follow a flow for Fig 2. For instance, where is proteostasis in Fig 2? It is a section in the discussion but not really mentioned in the figure. Also, Complement Activation is mentioned in Discussion, and is #3 cluster (Red) in Fig. 2. Please clarify the logic for the Discussion. It may be helpful to allude to the pathways based on Fig 2 (or respective figure) along with the cluster #. Another example is APOE and Lipid Metabolism, which seems to potentially come from Cluster # 7 (purple, lipoprotein regulation) in Fig. 2; in that case, it may help to use consistent subheading with the cluster name or annotate/explain the location of the corresponding figure.

We appreciate the reviewer’s feedback. **Fig. 2** only serves to demonstrate the identified pathways, we did not use it as the framework of our discussion. Instead, we prefer to discuss closely related clusters together, such as Proteostasis, which is a class of multiple pathways, as shown in **Table 1**.

We’ve changed the subheading to “Lipoprotein Regulation” as suggested.

18. It may help to really emphasize key findings in a paragraph in “Summary of Molecular Pathways in AD” as there is a lot going on in this paper (which is all great work), but the reader may be left wondering what to fully take away from this study. For instance, is this an approach that can be used in other diseases/contexts? Is this shedding light on future research in this area and where do we go from here? What can biologists grasp from these modules and findings? What is the key importance of these 5 classes of functionally-related molecular pathways that are significantly associated with AD? These findings are useful but it felt like they were not presented to the reader with enough justification of their importance. What is the future work?

We thank the reviewer’s suggestion, and have added a paragraph:

***pp. 18, para. 4***: “The above results demonstrate a landscape of AD etiology, and major pathological changes that range from molecular (gene transcription and RNA metabolism, proteostasis, and substance metabolism), cellular (immune reactions) to tissue-level (synapses) dysfunction. This approach can be applied to other complex diseases with significant genetic component. Future studies on modification and verification of the disease network may provide further insight on prevention and therapeutic intervention of AD.”

Minors
19. Grammatical errors and typos:
○ For example, under “Summary of Molecular Pathways in AD”, the first sentence contains an error, where “of” should probably be removed.
○ Another is in the introduction for the sentence: “We the integrate data of transcriptomic dysregulation in AD brains with the expanded AD disease module…”. Here, “the” should be replaced with “then”
○ In Fig. 5B, “Endothelia” could be a typo for “Endothelial”. If not enough space, perhaps abbreviating the name can help.
○ In Fig 3. Perhaps the x-axis label could be “log(OR) +/- 95% CI” with the / added between + and -.
○ Under Summary of Molecular Pathways in AD, 1st sentence may need hyphen “-” between functionally related, so it is: functionally-related

We apologize for the typos and have revised the main text and figures accordingly. We’ve also thoroughly checked the main text and corrected any mistake found.

20. Fig. 1 caption only mentions 2 words (Analysis workflow) and it may help to paraphrase the figure and the connections again so that the reader can recall the workflow. It may make the figure friendlier for readers then, as some could rely on the caption to help summarize the figure.

We’ve elaborated the figure legend of Fig. 1 as: “**Fig. 1: Analysis workflow.** 142 GWAS-identified AD genes were included as the seed genes of the AD disease module, and propagated to an expanded network consisting of another 646 predicted proximal genes based on the background protein-protein interactome (STRING). The expanded AD module was used for further functional annotation, pathway clustering and gene expression analyses integrating with transcriptomic data.”

21. In the introduction, it is mentioned that the latest large AD GWAS have identified over 40 risk loci. It is unclear if this is 1 GWAS or multiple GWAS (i.e. Jansen et. al, Kunkle et. al, etc.) SNPs combined. There was also a recent AD GWAS that has been published. The authors had 7 different references to GWAS studies for AD for this sentence, making it a bit confusing. It may help to refer to AD GWAS as “Jansen et. al”, “Kunkle et. al”, for instance. Again, a minor point, but it may be informative for readers.

We apologize for the confusion, and we meant to refer to multiple GWA studies, not a single GWAS. We’ve revised this sentence as “The latest large AD GWA studies have identified over 40 risk loci (3–9).”

22. Figure 3: it is really great and a reader-friendly figure with few key terms, which is really thoughtful. Nonetheless, it seems as though abnormal synaptic transmission and abnormal CNS synaptic transmission are very similar (even visually, in terms of log(Odds Ratio) +/- 95% CI) and from the terms, perhaps 1 of the terms is a subset of the other. It may be better to include another term instead or remove altogether or provide more context on why both were included.

These two terms were from the 10 most significantly enriched brain-related terms in the mammalian phenotype ontology (MPO) database. As data-driven results, we acknowledge the redundancy between these two terms, but feel obliged to report the results as they were, as it might be hard for readers to follow if we manipulate them.

23. This may be minor, but the number selected for random samplings could be consistent across approaches or explained more. For instance, why were 5,000 gene sets randomly selected to build up the null distribution for Network Propagation but only 1,000 random samplings used to build up the distribution for network localization? Is it due to computational resource constraints?

We thank the reviewer for pointing out this inconsistency. We have updated the localization results to be built on 5,000 random samplings.

24. Under network propagation, is z > 2 meant to be z > 1.96 (significance level of 5%) given that these are network propagation z-scores? Otherwise, what is the intuition for the cut-off of z > 2 for chance?

We agree with the reviewer that z > 1.96 would indicate a significance level of 5%. Here we chose z>2 to select genes which are greater than 2 standard deviations away from the mean. We have added a sentence to the text to provide more context for this choice.

***pp. 21, para. 3***: “…defined as 2 standard deviations from the mean (z > 2).”

25. In the Fig. S2 heatmap, the clustering and labeling is great, but it is a bit difficult to read the gene names properly. More height for the heatmap would help. A great resource in R could be the complexHeatmap package, which can really make beautiful heatmaps with annotations and clustering as well. Moreover, complexHeatmap could make the division in the heatmap between healthy and controls more prominent and easier to visualize.

We thank the reviewer for these suggestions and have updated Fig. S2 heatmap for better visualization of gene names.

26. Sharing the code on github or a publicly-available location can greatly help.

We’ve included the code on zenodo:

https://zenodo.org/record/5786722#.Ybtti73MKC8 (DOI: 10.5281/zenodo.5786722)

27. Instead of listing the URL for Agora directly (in section: “Transcriptomic dysregulation proximal to AD genes”), it may help to just cite it as the authors provide the full URL elsewhere in the methods.

We’ve removed the URL in Results as suggested. As no citation was suggested by the website, we simply describe about the database in Methods.

28. May be helpful to provide a citation for “Involvement of the cholinergic system in AD has been known since the 1970s”.

We’ve added the citation.

29. May also consider adding in GWAS p-values if it helps motivate findings.

We appreciate the reviewer’s suggestion. However, our **supplementary Table S1** includes both seed genes (from GWAS) and predicted proximal genes. As there are no p-values for proximal genes, it may look confusing for readers with only seed gene p-values added in.

**Reviewer #3**: The paper utilized a network propagation methods to build AD disease module with GWAS inputs. After that combing with transcriptome data, the authors proved that the identified AD disease module are enriched with multiple disease-associated pathways. And cell-specific expression data demonstrated that genes in AD disease module are enriched with microglia expressed immune and complement activation functions related genes.

I think the overall manuscript design is rigorous and systematic.

1. At the end of page 21 and beginning of page 22, for the network propagation formula, there is a parameter alpha in equation, can the author provides some metric to explain which alpha value you use and why you use this alpha value?

We have added this information to the methods:

***pp. 21, para. 2***: “We chose an α value of 0.5, based on previous work (*Vanunu et al. 2010, PMID: 20090828 PMCID: PMC2797085 DOI: 10.1371/journal.pcbi.1000641*) which demonstrated that the propagation algorithm is not sensitive to the choice of α as long as α ≥ 0.5.”

2. Second paragraph of page 22: node z score computation, in the method description, the authors mentioned node z score, however do NOT mention the corresponding p value, has the author considered p value as well or not (just z score)?

We have added the following text to the methods to indicate the corresponding p-value.

***pp. 21, para. 3***: “We compared the network propagation z-scores to a null model to find genes which were significantly more proximal to the seed genes than would be expected by chance, defined as 2 standard deviations from the mean (z > 2), where a z score of 2 corresponds to *p* = 0.02.”

3. The final formed AD disease module includes 788 genes with 142 risk genes and 646 network-proximity genes (non-GWAS evidenced). The authors have systematically discussed the 142 risk genes, however do not talk too much about the rest 646 network proximity genes. The authors could provide some statistical evidences for these 646 network-proximity genes by utilizing some public available AD-knowledge database, e.g., the open target platform, DisGeNET, to demonstrate that compared to randomly selected 646 genes, the 646 genes you predicted are more AD-associated. in this way, it could make people better believe that your method does have the capability to predict potential or likely AD-associated risk genes from GWAS inputs or the network module you found could provide useful information. I just think this could also help improve your manuscript novelties.

We appreciate the reviewer’s comment, and performed gene-disease enrichment analysis using DisGeNET (disgenet2r package for R). As expected, Alzheimer’s disease was found the top disease that the 646 proximity genes enriched. We added **supplementary Fig. S6**, and have added this new analysis to the methods and results.

***pp. 8, para. 1***: “Using the 646 proximal genes alone, the gene-disease enrichment analysis also demonstrated that AD was the top enriched disease (Fig. S6).”

***pp. 23, para. 1***: “We also evaluated the association of predicted 646 proximal genes with AD by gene-disease enrichment analysis (DisGeNET), using disgenet2r package for R.”

We would also like to clarify that we discussed both seed and proximal genes as risk genes based on their clusters.

**Have the authors made all data and (if applicable) computational code underlying the findings in their manuscript fully available?**
The [PLOS Data policy](https://urldefense.com/v3/__https:/journals.plos.org/ploscompbiol/s/materials-and-software-sharing__;!!LLK065n_VXAQ!z_7XhLPSfv27studTFAfNTadW3qrqmXY63K2MspuMah_kgLQ8llLA3JTSuowKUYLOQU$) requires authors to make all data and code underlying the findings described in their manuscript fully available without restriction, with rare exception (please refer to the Data Availability Statement in the manuscript PDF file). The data and code should be provided as part of the manuscript or its supporting information, or deposited to a public repository. For example, in addition to summary statistics, the data points behind means, medians and variance measures should be available. If there are restrictions on publicly sharing data or code —e.g. participant privacy or use of data from a third party—those must be specified.

Reviewer #1: Yes

Reviewer #2: **No:** open-source codes are missing for reproducibility

Reviewer #3: **No:** no code provided

PLOS authors have the option to publish the peer review history of their article ([what does this mean?](https://urldefense.com/v3/__https:/journals.plos.org/ploscompbiol/s/editorial-and-peer-review-process*loc-peer-review-history__;Iw!!LLK065n_VXAQ!z_7XhLPSfv27studTFAfNTadW3qrqmXY63K2MspuMah_kgLQ8llLA3JTSuowUtv7MCw$)). If published, this will include your full peer review and any attached files.


If you choose “no”, your identity will remain anonymous but your review may still be made public.


**Do you want your identity to be public for this peer review?** For information about this choice, including consent withdrawal, please see our [Privacy Policy](https://urldefense.com/v3/__https:/www.plos.org/privacy-policy__;!!LLK065n_VXAQ!z_7XhLPSfv27studTFAfNTadW3qrqmXY63K2MspuMah_kgLQ8llLA3JTSuowGvp2c7A$).

Reviewer #1: No

Reviewer #2: No

Reviewer #3: No

Figure Files:

While revising your submission, please upload your figure files to the Preflight Analysis and Conversion Engine (PACE) digital diagnostic tool, [https://pacev2.apexcovantage.com](https://urldefense.com/v3/__https:/pacev2.apexcovantage.com/__;!!LLK065n_VXAQ!z_7XhLPSfv27studTFAfNTadW3qrqmXY63K2MspuMah_kgLQ8llLA3JTSuowSz0AR30$). PACE helps ensure that figures meet PLOS requirements. To use PACE, you must first register as a user. Then, login and navigate to the UPLOAD tab, where you will find detailed instructions on how to use the tool. If you encounter any issues or have any questions when using PACE, please email us at [figures@plos.org](mailto:figures@plos.org).

Data Requirements:

Please note that, as a condition of publication, PLOS' data policy requires that you make available all data used to draw the conclusions outlined in your manuscript. Data must be deposited in an appropriate repository, included within the body of the manuscript, or uploaded as supporting information. This includes all numerical values that were used to generate graphs, histograms etc.. For an example in PLOS Biology see here: [http://www.plosbiology.org/article/info%3Adoi%2F10.1371%2Fjournal.pbio.1001908#s5](https://urldefense.com/v3/__http:/www.plosbiology.org/article/info*3Adoi*2F10.1371*2Fjournal.pbio.1001908*s5__;JSUlIw!!LLK065n_VXAQ!z_7XhLPSfv27studTFAfNTadW3qrqmXY63K2MspuMah_kgLQ8llLA3JTSuowKe5aQmc$).

Reproducibility:

To enhance the reproducibility of your results, we recommend that you deposit your laboratory protocols in protocols.io, where a protocol can be assigned its own identifier (DOI) such that it can be cited independently in the future. Additionally, PLOS ONE offers an option to publish peer-reviewed clinical study protocols. Read more information on sharing protocols at [https://plos.org/protocols?utm_medium=editorial-email&utm_source=authorletters&utm_campaign=protocols](https://urldefense.com/v3/__https:/plos.org/protocols?utm_medium=editorial-email&utm_source=authorletters&utm_campaign=protocols__;!!LLK065n_VXAQ!z_7XhLPSfv27studTFAfNTadW3qrqmXY63K2MspuMah_kgLQ8llLA3JTSuowlg4UEQY$)
