# Supplementary material for: Mapping the gene network landscape of Alzheimer’s disease through integrating genomics and transcriptomics
Source: PLoS Comput Biol. 2022 Feb 25;18(2):e1009903. doi: 10.1371/journal.pcbi.1009903 (PMC8906581; doi:10.1371/journal.pcbi.1009903)
Supplement: Attachment — Submitted filename: PLOS_Comput_Biol_response_2.docx [file pcbi.1009903.s004.docx]

Please take careful the minor comments from the Reviewer 2# and try to submit the revised manuscript in 2-4 weeks.

Reviewer's Responses to Questions

**Comments to the Authors:
Please note here if the review is uploaded as an attachment.**

**Reviewer #1**: The responses from authors have answered my questions well.

**Reviewer #2**: The authors have addressed most of my concerns. I just have some suggestions as follows.

6. The remark by the authors to point 1b is helpful and it is duly noted that presenting an alternative network could complicate the paper. Perhaps the limitations section could incorporate this limiting factor and mention in future work other networks could be considered. The authors could still reiterate the power of the STRING interactome as being the most robust interactome while still presenting the limitations of the approach.

We appreciate the reviewer’s suggestion, and added it in the main text:

***pp. 19, para 2***: “We chose the STRING interactome based on the results of a systemic evaluation of the performance of diverse networks (70), although other interactomes may yield different results and should be considered in future studies.”

7. It is great that the authors added in a paragraph describing the network propagation and what. It is helpful to perhaps elaborate a little further on what it means for heat to diffuse through a network, as that may be unclear still; at the moment, it sounds like a physics scenario with terms like heat, diffusion, etc. Nonetheless, it is great that the authors provided this helpful paragraph.

We thank the reviewer for the suggestion, and revised it in the main text:

***pp. 20, para. 3***: “The network propagation algorithm simulates how heat would spread, starting from a set of ‘hot’ seed genes (GWAS-discovered seed nodes). In the simulation, the heat spreads from gene to gene along the interactions in the network. The result is a set of ‘hot’ genes, which are likely related to the starting seed genes in biological process or pathway, as the adjacent genes in a network likely have similar biological functions. This process enables identification of genes related to multiple seed genes rather than a single one, because more heat will accumulate in genes that are close to multiple seed genes.”

9. Revision made to the figure caption. Nonetheless, it may help to add these same comments and clarifications (e.g. what was mentioned about Microglia and “immune reactions” and “substance metabolism”) to the figure text or the manuscript (or some supplementary documentation) as this clarification could help potential readers better understand the plot.

We appreciate the reviewer’s suggestion, and added the clarifying text in the figure legend.

***Legend of Fig. 5***: “… B) Classes of identified pathways that are functionally related are presented as inner circles, with circle sizes schematically but not quantitatively reflecting relative class sizes. The overlying outer circle illustrates the types of cells in the central nervous system with overall preferential gene expression in each major class (e.g., microglia overlay the classes of Immune reactions and Substance metabolism because genes in multiple clusters of these two classes were highly expressed in microglia).”

**Reviewer #3**: I checked my review feedback modifications; I am satisfied with all answers. Great work, I also learned multiple things.
